# 3D Printing of Shiitake Mushroom Incorporated with Gums as Dysphagia Diet

**DOI:** 10.3390/foods10092189

**Published:** 2021-09-15

**Authors:** Zhenbin Liu, Bhesh Bhandari, Chaofan Guo, Wenqi Zheng, Shangqiao Cao, Hongyu Lu, Haizhen Mo, Hongbo Li

**Affiliations:** 1 School of Food and Biological Engineering, Shaanxi University of Science and Technology, Xi’an 710021, China; zhenbinliu@sust.edu.cn (Z.L.); zhengwenqifcns@163.com (W.Z.); caoshangqiao2021@163.com (S.C.); hongbo715@163.com (H.L.); 2 State Key Laboratory of Food Science and Technology, Jiangnan University, Wuxi 214122, China; guochaofanfan@outlook.com; 3 School of Agriculture and Food Sciences, The University of Queensland, Brisbane, QLD 4072, Australia; b.bhandari@uq.edu.au; 4 Jiangsu Provincial Supervising and Testing Research Institute for Products’ Quality, Nanjing 210000, China; hyluuuuu@126.com

**Keywords:** 3D printing, shiitake mushroom, dysphagia, IDDSI

## Abstract

With the speeding tendency of aging society, the population experienced dysphagia is increasing quickly. Desirable dysphagic diets should be safe, visually appealing and nutritious. 3D printing allows for creation of personalized nutritious foods with regular-like appearance. Shiitake mushroom, rich in protein and bioactive compounds, is suitable for elderly, but its hard texture was not friendly to the elderly with dysphagia. This study investigated the feasibility of production of dysphagic product using shiitake mushroom by 3D printing with various gums addition, including arabic gum (AG), xanthan gum (XG) and *k*-carrageenan gum (KG) at concentrations of 0.3%, 0.6% and 0.9% (*w*/*w*). Data suggested that XG and KG incorporation significantly increased inks’ mechanical strength by decreasing water mobility and promoting the formation of hydrogen bond, enabling 3D printed objects with great self-supporting capacity. The XG containing and KG-0.3% samples were categorized into level 5—minced and moist dysphagia diet within international dysphagia diet standardization initiative (IDDSI) framework. AG addition decreased mechanical strength and viscosity, hardness and self-supporting capacity of 3D printed constructions. AG-0.3% and AG-0.6% samples could not be classified as dysphagia diets based on IDDSI tests. This study provides useful information for dysphagia diet development with appealing appearance by 3D printing.

## 1. Introduction

With the rising aging population trend, the proportion of elderly suffering from weakness of chewing and swallowing is increasing rapidly. Dysphagia refers to the swallowing difficulty, displaying an abnormal delay in food bolus movement during swallowing, which would easily cause coughing, choking, or difficulty of swallow initiation due to the food residue left in oral cavity [1]. The people with chewing or swallowing problems are prone to nutritional deficiency and weight loss because of less food consumption. To prevent malnutrition or weight loss of such population, foods should be soft and moist enough to chew and swallow safely. Therefore, the foods for the chew- or swallow-impaired individuals are usually in pureed or mashed state [2,3]. However, these pureed or mashed foods are not appetizing and visually unappealing, less palatable and nutritionally diluted. In addition, the meal choices for dysphagia patients are fewer with poor meal presentation. Therefore, supplying dysphagia patients with nutritious and safe foods imposes a great challenge [4,5]. Efforts must be made to provide regular-like meals to enhance the willingness to consume.

The current method of using mold to shape pureed foods can create aesthetically pleasing dishes, but it requires a special training and cannot modify food texture as required. 3D food printing can create the foods through layer by layer manner based on digital designs. The benefits of 3D food printing also include nutrition personalization, custom designing based on special mealtime needs, production of visually appealing foods, and decrease in requirement of personnel skills [6,7]. 3D food printing can provide an excellent solution to the preparation of visually appealing, and texturally modified pureed foods, which are very friendly to dysphagic patients. Recently, the creation of dysphagia-oriented dies using 3D printing has raised researcher’s attention, but needs to be improved further. To our knowledge, only four publications on this subject have been reported, which were based on meat or vegetable ingredients. Specifically, 3D printed pork and beef products were classified as potential dysphagia food [8,9]. Tuna pureed [10] and vegetable puree [11] were also used for the creation of dysphagia diets using 3D printing. More efforts should be made on the production of dysphagia diets by 3D printing with more varieties of food materials.

Shiitake (*Lentinus edodes*), which accounts for 17% of the global edible fungi [12], is the second most consumed mushroom in the world due to its good texture, nutritious effects. In addition, it illustrates many health promotion effects, such as anti-oxidant and reducing hypercholesterolemia properties, which probably attributed to the ingredients of lentinan (β-1,3-glucan) [13,14]. Shiitake is rich in protein, but low in fat, which is very suitable for the elderly as a nutritionally rich diet [13,14]. However, its hard texture and requirement of great chewing efforts makes it difficult for the elderly or dysphagia patients. Thus, it would be meaningful to process shiitake (*Lentinus edodes*) into palatable appetizing food that is easy to chew and swallow.

This study aims to develop shiitake dysphagia diets, based on shiitake as the main ingredient by 3D printing with appealing appearance.

## 2. Materials and Methods

### 2.1. Experimental Design

To achieve desired 3D printing performance and chewing/swallowing properties, various gums (XG, KG and AG) were added and resultant rheological properties were characterized, and LF-NMR and FTIR were then conducted to investigate the possible mechanism. 3D printed samples were finally evaluated in terms of texture properties and IDDSI recommended tests to determine their suitability for dysphagia patients. The test was conducted in two or three replicates, and the average value was reported.

### 2.2. Materials

Shiitake mushroom powder was purchased from Bao-feng biological science and technology Ltd., Anhui, China. Gums of XG, AG and KG, in food grade, were obtained from Usolf Chemical Technology Co., Ltd., Qingdao, China. Same batch of mushroom powder and gums were used throughout the experiment.

### 2.3. Preparation of Shiitake/Gum Mixed Ink for 3D Printing

Shiitake powder was mixed thoroughly with each of the above-mentioned gums at 0.3%, 0.6% or 0.9% (*w*/*w*) to avoid the aggregation while dissolving in water. Deionized water (3 times the weight of mixed powder, according to preliminary tests) was then added to the powder, which was then homogenized for 3 min at 1000 r/s by using a homogenizer (T18BS25, IKA instrument equipment Co., Ltd, Guangzhou, China) to homogenize the mixtures. Next, the mixtures were heated at 85 ± 0.2 °C for 30 min using water bath after covering the container with a thin layer of cling wrap to avoid the moisture loss or water gain during cooking process. Finally, the ink samples were kept at 25 °C for 3 h prior to use. The ink samples with 0.3, 0.6, and 0.9% of arabic-, xanthan- and *k*-carrageenan-gum were respectively labeled as AG-0.3%, AG-0.6%, and AG-0.9%; XG-0.3%, XG-0.6%, and XG-0.9%; and KG-0.3%, KG-0.6%, and KG-0.9%. The sample without gum was labeled as control.

### 2.4. 3D Printing

An extrusion-based dual nozzle printer manufactured by Shiyin Technology Co. Ltd., Hangzhou, China, was used in this study. For detailed information about the printer, reader can refer to our previous study [15]. Printing and environment were both at 25 °C, and only one nozzle was used. Printing parameters were optimized through preliminary experiment as following: nozzle diameter of 1.2 mm, layer height of 1.2 mm, printing speed of 25 mm/s, infill pattern of rectilinear, infill level of 100%, perimeter of 2 layers. A hollow cylinder (28 mm outer diameter, 18 mm inner diameter 18 mm, and 20 mm height) was printed to assess ink’s printability. A cuboid shape (edge length of 15 mm) was printed for texture analysis and fork pressure test. Additionally, several complex structures were also printed.

### 2.5. Rheology Tests

Rheological properties of ink samples were tested using a rheometer (AR-2000 ex, TA Co. Ltd., New Castle, DE, USA). A 40 mm diameter steel plate geometry was used with a 1000 μm gap. During the test, the trap was covered with a thin oil layer to avoid the water loss. Before each test, the ink samples were equilibrated for 5 min at test temperature to reach a steady state. Viscosity properties of ink sample was determined with shear rate increased from 0.1 to 100 1/s. Yielding behavior of ink samples was conducted by oscillation stress sweep test with stress varying from 1 to 2000 Pa at 1 Hz. Oscillation strain sweep test was conducted within 0.01–100% strain at 1 Hz. Creep measurement was performed with a 10 Pa stress for 600 s, to determine the deformation resistance of ink samples. Unless otherwise stated, the temperature was remained at 25 °C. Two replications were measured.

### 2.6. Low Field Nuclear Magnetic Resonance (LF-NMR) Measurement

A LF-NMR analyzer produced by Numag Electric Co., Suzhou, China was used to test control and 0.6% gum containing ink samples’ relaxation time T_2_ at a magnetic field intensity of 0.5 T and a constant temperature of 32 °C, respectively. Before test, oil sample was firstly used to calibrate the analyzer. The built-in Carr-Purcell-Meiboom-Gill procedure was then applied to analysis the water mobility. Every measurement around 5 g of ink sample was wrapped with a thin cling wrap before placing into the measurement. Test parameters were selected as following: sampling point (TD) = 59,992, spectral width (SW) = 100 kHz, Echo Count (NECH) = 3000, number of scan repetition (NS) = 4, sampling repetition time (TW) = 3000 ms. Three replications were measured.

### 2.7. Fourier Transforms Infrared (FTIR) Analysis

An FTIR spectrometer (Thermo Co. Ltd., Massachusetts, America) was applied to determine spectral absorption characteristics of control and 0.6% gum containing ink samples. Ink samples were firstly freeze-dried and made into fine powder, mixed with KBr powder (spectroscopic grade), and then pressed into 1 mm pellets (sample:KBr = 1:100). The spectra curve was performed within the frequency range of 4000 to 400 cm^−1^ at 25 °C. Three replications were conducted.

### 2.8. Texture Profile Analysis (TPA)

Texture properties of 3D printed samples were conducted using a texture analyzer (Stable Micro System Ltd., Leicestershire, UK). Probe P/75 with a diameter of 75 mm, which was greater than the printed samples, was used to test the force-distance graphs. Texture analyzer was firstly calibrated, followed by the height calibration. During TPA test, two cycles of compression was conducted. When the first compression completed, the plunger reversed at 2 mm/s, and a second compression began after holding for 5 s. The printed cuboid shape (edge length 15 mm) sample was attached to the platform’s center. Measurement was performed as follows: pre-test rate 5.0 mm/s, test and post-test rate 2.0 mm/s, holding time 5 s, trigger force 10 g, compression strain 45%, and test temperature at room temperature (25 °C). Three replications were measured. Three replications were conducted.

### 2.9. International Dysphagia Diets Standardization Initiative (IDDSI) Test

The drinks and foods suitable for dysphagia patients are divided into 8 categories, 5 levels of drinks and 5 levels of foods, in which transitional foods including 3 levels (level 5-minced & moist; level 6-soft & bite-sized; level 7-regular or easy to chew). Level 4 is used to describe both drink (extremely thick) and foods (puree). Based on preliminary tests, the ink samples used in this study was probably categorized into level 4 or level 5 within IDDSI framework, and detailed description of these two levels of foods is listed in Table 1 [16]. The sample was tested according to IDDSI methods to evaluate whether each sample could be qualitatively measured and classified within IDDSI framework. The ink sample before 3D printing was evaluated by fork drip test and spoon tilt test. During fork drip test, the ink samples were determined by scooping them up with the fork and observing their flow behavior through the prongs, which was compared with the descriptions of foods of each level within IDDSI. Spoon tilt test was conducted as follows: scoop a teaspoon full of sample, hold the spoon steady above a plate, then tilt the spoon sideways slowly. During this process, the ink’s behavior was compared with IDDSI descriptions. Additionally, 3D printed samples with the size of 1.5 × 1.5 × 1.5 cm^3^ was selected for fork pressure test. During fork pressure test, a fork with pressure was applied on the printed samples to observe their behavior.

### 2.10. Statistical Analysis

The data reported in this research correspond to average values with standard deviation. One-way ANOVA method with 95% confidence level was applied. Duncan’s test with *p* < 0.05 was considered to be significant difference between samples using SPSS 22 (IBM SPSS Statistics, Chicago, IL, USA). Graphs were plot using Origin 2018 (OriginLab, Northampton, MA, USA).

## 3. Results and Discussion

### 3.1. Rheological Properties

Rheological properties are crucial important for 3D printing performance and suitability for dysphagia diet. Currently, 3D printing behavior has been reported to be closely related with rheological properties. Yield stress and viscosity greatly affected the extrusion behavior of food inks during printing [17]. Self-supportable performance of 3D printed construction was highly related to G′ and phase angle of food inks [8,9]. As for dysphagia-oriented or swallowed-impaired diets development, the importance of designing rheological properties has also been stressed. In particular, the rheological parameters, such as viscosity, viscoelastic behavior and yielding property, are important for swallowing process [18,19]. The requirement for safe swallowing is the balance among the rheology of food bolus, driving strength imposed by oropharyngeal muscles to protect the airway. Rheological properties are believed to be the most important aspect of dysphagia-oriented diets essentially to achieve such balance [20]. Therefore, in this work rheological properties of ink samples were investigated and discussed in relation to 3D printing behavior and chewing/swallowing properties.

#### 3.1.1. Yield Behavior

**Figure 1:**
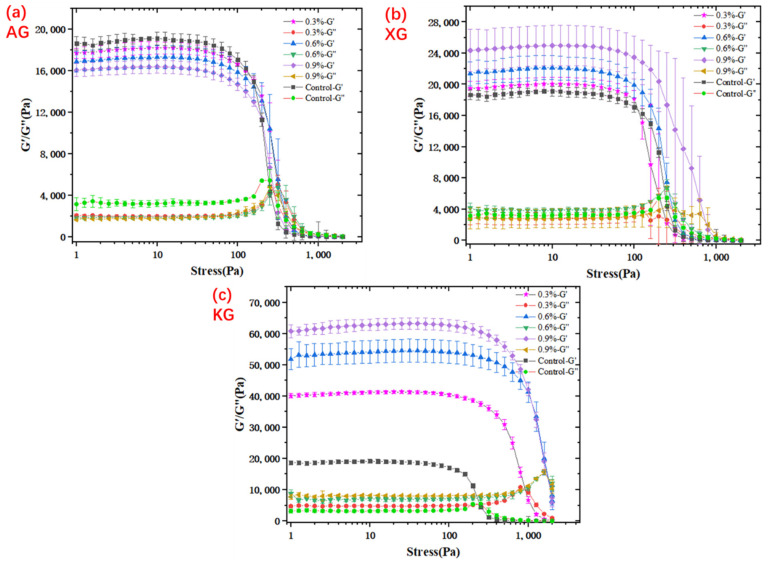
Oscillation stress profiles of ink samples: (**a**) AG; (**b**) XG; (**c**) KG.

Yield stress is critical in determining flow’s extrusion behavior during 3D printing as it indicates the minimum force required to start the ink flow, below which the ink cannot flow and exhibits solid-like property [17]. In extrusion-based printing, ink with a low yield stress is desired because the extrusion process is not continuous; rather it starts and stops frequently as designed. An ink with a high yield stress needs printer to generate a larger pressure to enable the smooth extrusion, which was not easy for the extrusion unit to constantly generate such a high force in a controlled manner [17]. For dysphagia-oriented diets, yield stress has also been reported to be an important “rheological criterion” [21]. It relates to the minimum stress to start a flow, below which the food bolus cannot be moved by tongue. Thus, it is critical to determine yield stress of foods to be swallowed [21]. Figure 1 illustrates stress profiles of ink samples with various amount of added gums. When the oscillation stress increased, a plateau constant value of G′ and G″ were observed firstly, and then G′ decreased sharply while G″ increased rapidly until it intersected with G′. The intersection points where G′ equals to G″ is defined as yield stress [17]. Generally, addition of KG and XG increased the plateau constant values of G′, G″ and yield stress. Particularly, yield stress was greatly improved when the amount of XG increased from 0.3% (268 Pa) to 0.9% (751 Pa). This was possibly because XG molecules could transform from a disordered state to an ordered structure through intermolecular associations, resulting in a network structure. Obviously, a higher XG content is easier to form a stronger network structure [22,23]. As for the ink samples containing KG, yield stress significantly increased from 941 Pa to 1803 Pa when KG increased from 0.3% to 0.9%. Moreover, in comparison with XG and AG, KG addition significantly improved the yield stress of ink samples. Specially, when the hydrocolloid amount was 0.9%, the yield stress of KG sample was 1803 Pa, which is 2.40 times of XG sample (751 Pa). This can be attributed to the formation of carrageenan double helices firstly, followed by their aggregation and finally forming a strong gel network structure with strong mechanical strength [24]. Theoretically, the anion-polysaccharide could interact with polymers by electrostatic and hydrogen bonds, improving the strength of gel structure [25,26]. However, AG addition decreased the plateau constant values of G′ and G″ compared with control sample (Figure 1), suggesting that AG decreased the mechanical strength of ink samples. This was probably because AG addition prevented the hydrogen bonds formation in the ink system, which was found to be consistent with FTIR results, which will be further discussed later. Consistently, Zhao et al. [26]. reported that the addition of AG decreased the gel strength and hardness of fish gelatin because AG prevents the formation of hydrogen bonds. Fang, et al. [27] also stated that AG addition weakened the elastic structures and gel strength of gelatinized waxy potato starch. Azam, et al. [28] suggested that AG addition decreased the apparent viscosity of starch-orange concentrate gel system because of the breakdown of its structure in the presence of the protons.

#### 3.1.2. Viscosity and Shear Thinning Behavior

Regarding 3D printing, after starting the flow, the viscosity profiles significantly affect the minimum force to keep the flow continuous. For a desirable food inks, viscosity should be low enough during extrusion to enable continuous flow through a thin nozzle tip and high enough to adhere with previously extruded layers. From this viewpoint, shear-thinning property with good shear-recovery ability is highly preferable [17]. Additionally, shear-thinning property was also reported favorable for dysphagia foods, as such property enable food bolus to keep in high viscosity at steady state but easy to swallow during chewing when shear force is rendered by the tongue [18].

**Figure 2:**
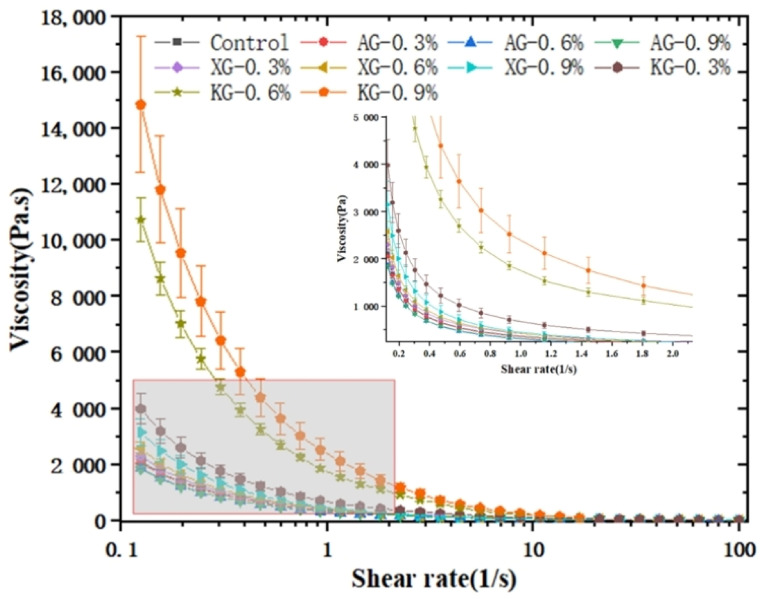
Apparent viscosity profiles for different ink samples.

Figure 2 illustrates the viscosity profiles of inks with or without the addition of gums. All ink samples showed shear-thinning property, which is characterized by a viscosity reduction with the increase of applied shear rate, and no Newtonian plateau was observed. A small amount of XG and KG addition obviously increased the viscosity of ink samples within the shear rate range of 0.1–10 1/s. When the shear rate was over 10 1/s, the viscosity behavior of ink samples tended to converge, which was close to zero. Specifically, as for XG-containing ink sample at 0.3% and 0.6%, the viscosity respectively rapidly decreased from 2304 Pa to 66.52 Pa and from 2579 Pa to 61.92 Pa when the shear rate increased from 0.1 to 10 1/s. Addition of KG significantly increased the viscosity of ink samples compared with XG and AG, when the shear rate was less than 10 1/s. Specifically, as seen from Table 2, at 1/s, the viscosity of KG-0.9% sample was 2122.33 Pa, which was 5.27 times of XG-0.9% sample (402.87 Pa) and 7.77 times of AG-0.9% sample (273.30 Pa). AG addition decreased the viscosity of ink samples, for example, at 1/s, the viscosity of AG-0.3%, AG-0.6% and AG-0.9% samples were 313.75 Pa, 289.40 Pa and 273.30 Pa, respectively (Table 1). Again, this can be probably attributed to the AG addition preventing the hydrogen bond formation as discussed above [26,27].

For further quantitative description of the shear-thinning behavior of ink samples, the power-law model ($\eta =K\gamma^{\left(n-1\right)}$) was applied. Where *ƞ* indicates apparent viscosity, *K* indicates consistency index, γ indicates shear rate, and *n* indicates flow index. A value of *n* lower than 1 indicates shear thinning property and a smaller *n* suggests a strong shear-thinning characteristic [17]. Table 2 displays the power-law fitted parameters for ink samples with different formulation. All the *n* of inks samples was lower than 1, which further reveals that ink samples were shear-thinning fluids. In addition, *n* decreased when each gum concentration increased, indicating that shear-thinning behavior was improved when gum concentration increased. Specifically, the *n* of KG-0.3%, KG-0.6%, and KG-0.9% was respectively 0.14, 0.12, 0.10. XG containing samples displayed relatively small *n* values ranging from 0.11 to 0.06 when XG concentration increased from 0.6% to 0.9% (Table 2). This was probably because the network structure formed by xanthan molecules was destroyed rapidly when shear force was applied [29,30]. It was also reported that the xanthan molecules aggregation and hydrogen bond formation account for the highly shear-thinning property of XG containing samples [18]. Shear-thinning characteristic is desirable during swallowing with reduced aspiration risk through providing neuromuscular system a longer reflex response time to close the epiglottis [18]. Moreover, samples with strong shear-thinning behavior present less slimy feeling during chewing, which can be a critical factor in providing the patients with a pleasure texture. From this viewpoint, compared with control sample (*n* value of 0.19), gum addition increased the suitability for dysphagia diets, indicated by the decrease in *n* value.

Consistency index *K*, closely correlated with extrusion performance of food inks during 3D printing, is shown in Table 2. Too high *K* would cause the extrusion difficulty and lines breakage of extruded inks, resulting in the failure of 3D printing [17]. KG addition significantly increased *K* from 670.97 Pa·s^n^ to 2242.43 Pa·s^n^ when its concentration increased from 0.3% to 0.9%. This was expected since a higher amount of KG is easier to form a denser network structure caused by the double helices aggregation [31]. Similar trend was observed for XG addition, the *K* of XG-0.3%, XG-0.6%, and XG-0.9% ink samples were 400.32 Pa·s^n^, 408.56 Pa·s^n^ and 469.94 Pa·s^n^, respectively. This was probably because the rigid ordered state of xanthan helix intermolecular associations leading to the formation of a network structure [29,30]. In contrast, the addition of AG decreased the *K* of ink samples. In particular, the *K* decreased from 347.17 Pa·s^n^ to 305.69 Pa·s^n^ when AG concentration increased from 0.3% to 0.9%. Again, the prevention of formation of hydrogen bonds might have contributed to this effect [26,27].

#### 3.1.3. Viscoelasticity Behavior

**Figure 3:**
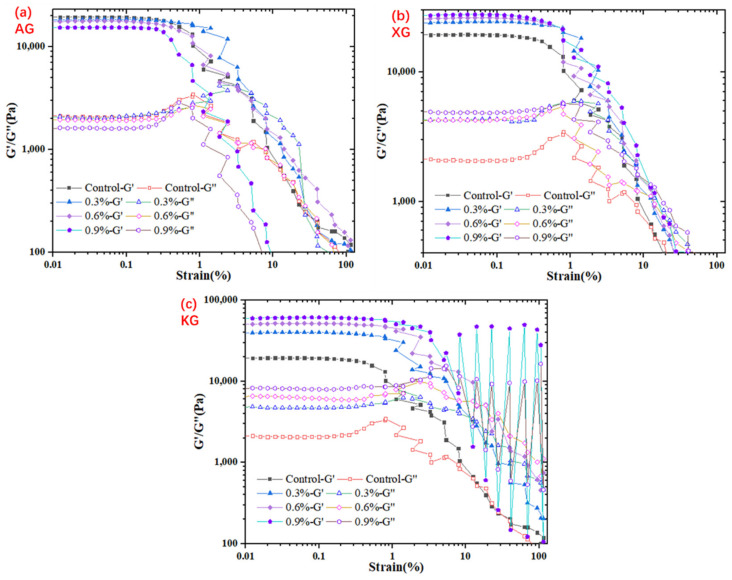
Strain-dependence of viscoelasticity of ink samples with different formulation. (**a**): AG; (**b**): XG; (**c**): KG.

Viscoelasticity behavior like G′ is important for self-supporting performance of printed samples. G′ indicates solid-like behavior reflecting the mechanical strength and deformation resistance ability [17]. Additionally, regarding swallowing behavior, viscoelasticity of food also reflects the overall effect on swallowing pleasure [18,32]. To study the viscoelasticity behavior of ink samples with different formulation, amplitude oscillation strain sweep test was conducted at 1 Hz with strain ranging from 0.01% to 100%, as shown in Figure 3. All ink samples illustrated a similar pattern, that is within linear viscoelastic region (LVR). Both G′ and G″ were nearly independent of strain with G′ greater than G″, suggesting a solid-like gel behavior. Once exceeding the critical strain, G′ decreased sharply while G″ increased rapidly until reaching a crossover point. After critical strain, G″ became greater than G′, which indicates that the destruction of gel network structure and beginning of flow. KG addition significantly increased G′ of ink samples within LVR, and a higher amount of KG led to a higher G′. For example, the average value of G′ (within LVR) increased from 39554 Pa to 60890 Pa when KG increased from 0.3% to 0.9%. This was because KG probably formed a network structure through the aggregation of double helices, and a higher concentration enabled an easier formation [31]. As expected, an increase of G′ was also observed in the XG added samples. This was because xanthan gum can form intermolecular associations resulting in the creation of a network structure of bound molecules [29,30]. Interestingly, it should be noted that the addition of AG generally decreased the plateau values of G′ and G″ within LVR (Figure 3). This was consistent with the results of yield behavior (Figure 1) and viscosity profiles (Table 2). Again, this might be because the AG addition prevented the hydrogen bond formation [26,27].

#### 3.1.4. Creep Deformation Behavior

**Figure 4:**
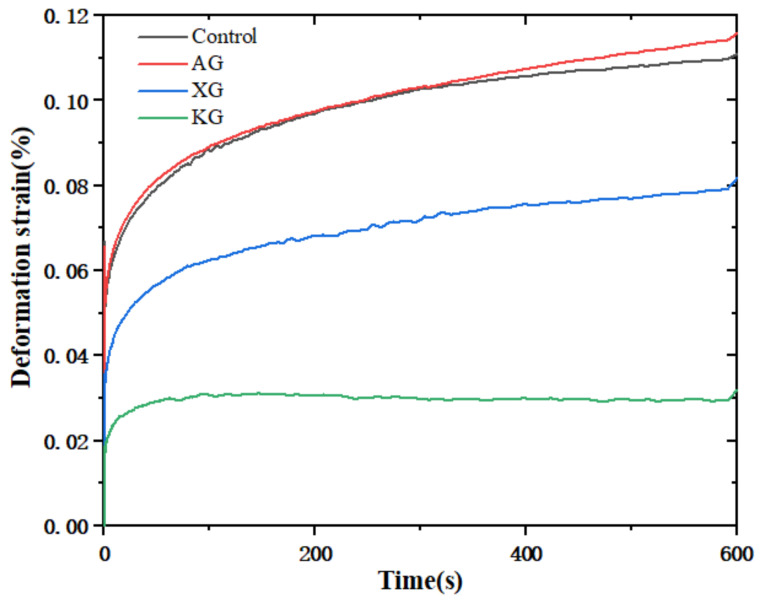
Creep response of ink samples with different formulation at a gum concentration of 0.6%.

Creep deformation performance can reflect the self-supporting capability of 3D printed object [33]. In addition, during swallowing process the tongue provides a constant driving pressure to enable food bolus pass to the throat. Food bolus’s deformation under certain constant stress is also an important factor for safe swallowing [18,20]. The creep deformation behavior of control and ink samples containing three kinds of gums is shown in Figure 4. Incorporation of KG and XG improved the deformation resistance capability, indicated by decreased maximum strain for KG (0.03%) and XG (0.08%) samples, compared with control (0.11%). The deformation resistance ability illustrates a decrease order of KG > XG > control > AG, consistent with the results of G′ and yield performance as reported above (Figure 1 and Figure 3). This suggested that although KG and XG containing sample possessed good resistance to compressed deformation during 3D printing, they also required a high-pressure application to deform the food bolus during swallowing, which was not actually friendly for the swallow-impaired patients.

### 3.2. Water Mobility Studied by LF-NMR

**Figure 5:**
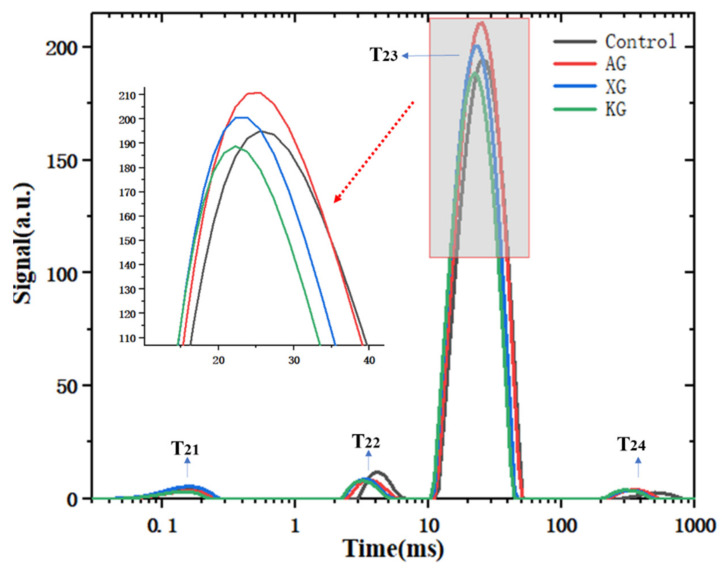
Relaxation time distribution for ink samples with different formulation at gum concentration of 0.6%.

Water molecules mobility correlates with material’s rheological properties. Previous studies reported that the formation of a strong gel network structure reduces the mobility of water molecules [34]. Figure 5 shows the relaxation time (T_2_) of ink sample with different formulations. The water population of T_21_ and T_22_, occurred at less than 10 ms, which represent the water fraction closely bound with biopolymers with less mobility. T_23_ population peaked between 22.22–25.53 ms, accounting for the maximum proportion (93.83–95.19%), reflects the more mobile water fraction. The T_24_ water population, with relaxation time greater than 100 ms, indicates the free water fraction with the greatest mobility [34]. T_23_ peak relaxation time shifted close to 0 ms for the samples containing KG (22.22 ms) and XG (23.82 ms) compared with control (25.53 ms). Moreover, the proportion of T_23_ population of KG (93.83%) and XG (93.86%) samples was lower than that of control sample (94.18%) (Figure 5). These facts suggested the presence of less mobile water due to the enhancement of network structure after KG and XG addition. This also explain the fact of greater G′ and *K* of XG and KG containing ink samples, as shown in Table 2 and Figure 3. This is apparently because water molecules are closely hydrogen bonded to macropolymers with less movement [33]. AG addition did not cause an obvious change of T_23_ peak time compared with control sample, which happened most likely because of the formation of low viscous gel by AG [28].

### 3.3. Intermolecular Interaction Analyzed by FTIR

**Figure 6:**
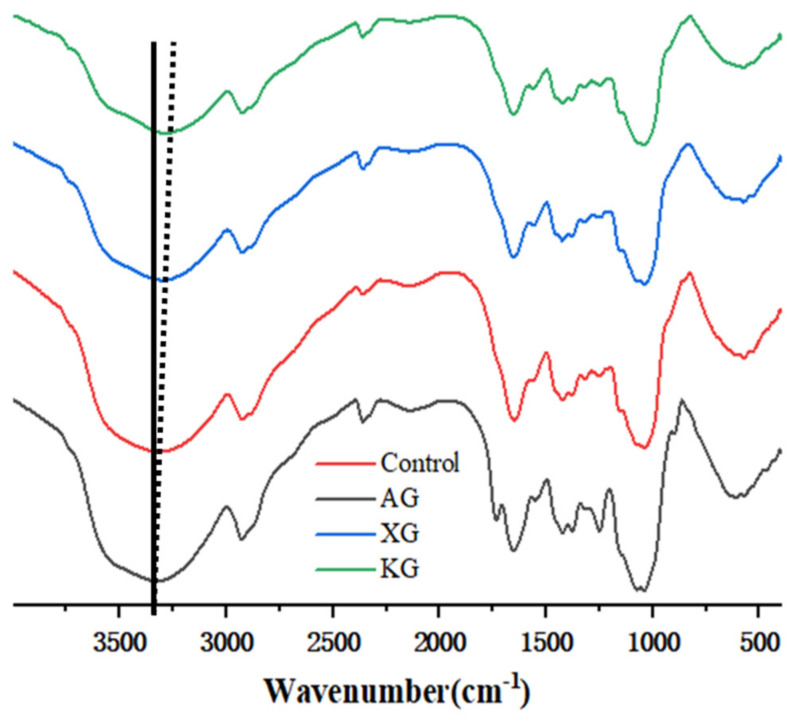
FTIR spectra for ink samples with different gum at concentration of 0.6%.

FTIR is an effective method to detect the presence of hydrogen bond and further compare their strength indirectly. A lower wave number indicates a stronger interaction among components. Samples with (0.6%) and without gums were analyzed using FTIR from 400 cm^−1^ to 4000 cm^−1^ (Figure 6). Significant absorptions at around 2930 cm^−1^, and 1730 cm^−1^ were observed, which are assigned to the asymmetric stretching vibration of methyl groups (C–H), and C=O stretching vibration (carbonyl or amide), respectively [35,36]. Absorption band at around 1050 cm^−1^ corresponds mainly to C-O and C-C stretching vibrations in polysaccharide [36,37,38]. Lentinan, the most important functional ingredient of shiitake, produces absorption band at 1650 cm^−1^ (C=O stretching) and 1400 cm^−1^ (carboxylate) [39]. As seen from Figure 6, the FTIR curves showed a comparable pattern, suggested that no new functional group was generated after gum added. There was a broadband absorption peak for all samples at around 3325 cm^−1^, which was ascribed to the vibrational stretching rising from molecular bound hydroxyl groups or absorption bands of poly-OH [23,36]. Particularly, the absorption broadband appeared at 3325 cm^−1^ for control sample, 3322 cm^−1^ for XG sample, 3316 cm^−1^ for KG sample, respectively. The decrease of wavenumber after addition of KG and XG indicated the enhancement of hydrogen bond formation. This might account for the greater G′ (Figure 1) and *K* (Table 2) of KG and XG added samples. In contrast, AG addition led to a slight shift toward longer wavenumber (3335 cm^−1^) compared with control and KG or XG added samples. This might be used to explain the decrease of mechanical strength (G′ and yielding behavior) of AG containing samples.

### 3.4. Texture Properties

**Figure 7:**
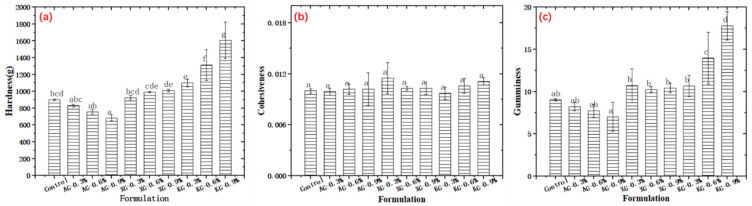
Texture properties of 3D printed samples. (**a**): hardness; (**b**): cohesiveness; (**c**): gumminess.

Figure 7 shows that the texture properties of 3D printed cuboid shape samples (edge length 15 mm) with different formulations. Here it should be kept in mind that a probe with a greater diameter (75 mm) than printed samples was applied. During test, it was observed that the samples would easily attach to the probe and transverse upward together with the probe during the first compression cycle. Thus, a thin plastic film was covered on top of the samples to avoid this adhesion [15], and as a result the adhesiveness result is not reported. Figure 7 shows that KG added samples had the highest hardness and gumminess, and a higher concentration led to higher values. In particular, when the KG concentration increased from 0.3% to 0.9%, the hardness and gumminess of samples respectively increased from 1100.43 g to 1606.47 g and from 10.68 to 17.81, compared with that of 899.67 g and 9.03 g of control samples. This was because a higher KG concentration is more easily to form a stronger network structure caused by the double helices aggregation [31]. Compared with control sample, XG addition slightly increased the hardness of samples, but with no significant changes in gumminess. This was because XG molecules could transform from a disordered structure with more flexible to a rigid ordered structure enhancing the mechanical strength of samples [29,30,40]. Exceptionally, for AG containing samples the hardness and gumminess were lower than that of control. This was probably because AG’s nature of forming low viscous gel and the prevention of forming hydrogen bonds [26,27]. Additionally, no differences were found for cohesiveness among samples, with relatively small values ranging from 0.010 to 0.012. Here, it should be noted that the cohesiveness obtained by TPA might not be reliable. In previous study, Nishinari, et al. [41] reported that the cohesiveness of water obtained by TPA was 1 while the yogurt was less than 1. This is not reasonable as it is well known that water is easy to induce aspiration because of its least cohesive nature and tends to scatter into smaller droplets [41]. It is well accepted that the ideal dysphagia diets should have low hardness, appropriate cohesiveness requiring minimal chewing effort. Such textural properties are correlated with the food bolus formation that allows the ease of swallowing initiation and swift transport through the pharynx [42]. From this viewpoint, the KG containing samples might not be feasible for swallow-impaired adults due to the high hardness and gumminess. However, it should be noted that only TPA test might not be reliable enough to determine the feasibility of dysphagia diets [41]. Thus, the following IDDSI tests were conducted.

### 3.5. IDDSI Tests

The International Dysphagia Diet Standardization Committee released the IDDSI framework in 2016, which consists of a continuum of 8 levels (0–7) to provide the global standardized terminology and definitions to describe the foods or liquids suitable for dysphagia diets [16]. IDDSI tests are convenient and widely used to evaluate whether certain foods are feasible for dysphagic patients. In this study, samples were evaluated using IDDSI methods of fork pressure test, fork drip test and spoon tilt test, to determine whether they can be categorized within IDDSI framework (Table 3). The fork drip test was applied on ink samples to determine whether they flow through the prongs of a fork. Results showed that all samples piled above the fork, and there was no sample flow or drip continuously through the fork prongs, consistent with the description of food level 5—minced and moist within IDDSI framework. The 3D printed samples with the size of 1.5 × 1.5 × 1.5 cm^3^ was used for the fork pressure test, because such size is believed to decrease choking risk [43]. The average food particles size before swallowing was 2~4 mm. The gap between prongs of a standard metal fork is usually 4 mm, providing an effective measure for the size of food particle. During the fork pressure test, the pressure conducted to make the thumb nail blanch was reported at around 17 kPa, which is consistent with tongue pressure applied during swallowing [44]. Results suggested that all 3D printed samples regardless of formulation were easily separated between and come through the prongs of the fork with no lumps and minimal granulation, and a clear pattern was made on the sample’s surface. Additionally, the printed samples were easily mashed and deformed with little pressure (not enough to make the thumb nail blanching to white), and they did not return to original shape after removing the fork. This indicated that all the samples passed the fork pressure test, complying with the criteria of level 5—minced and moist dysphagia food category [16]. The spoon tilt test, conducted by scooping sample onto the spoon and tilting the spoon to observe ink sample’s movement behavior, was used to evaluate ink sample’s stickiness and ability to hold together (cohesiveness). Results suggested that all the sample were cohesive enough to hold their shape on the spoon. The samples of AG (0.9%), XG (0.3%, 0.6%, 0.9%), KG (0.3%) and control easily slide off when the spoon was titled or turned sideways with the application of a very gentle flick, with little food left on the spoon. However, although the samples of AG (0.3%, 0.6%), KG (0.6%, 0.9%) passed the fork pressured and fork drip tests, they were not easy to slide off when the spoon was titled or turned sideways, with lots of food left on the spoon. This suggested that they did not meet any of the levels described under IDDSI framework since they were too sticky for levels 4 and 5, and too soft for levels 6 and 7. It has been reported that too adhesive foods were not safe for individuals with swallow-impairment, because it would increase chocking risk and require greater lingual effort to propel foods into and through the pharynx [42,45,46]. Generally, according to the IDDSI tests of spoon tilt, fork pressure and fork drip (Table 3), the samples AG (0.9%), XG (0.3%, 0.6%, 0.9%), KG (0.3%) and control were complying IDDSI level 5—minced and moist.

Here, please note that no sensory tests were conducted on 3D printed samples for dysphagia. This was because the inconsistent results for the individual’s perception of 3D printed foods, attributed to the fear of an emerging technology in contrast to its benefits [8,47]. Previous studies indicated that the swallowed-impaired adults’ acceptance of traditional pureed foods was similar or higher than molded foods [48]. This was probably because the taste alteration due to the requirement of hydrocolloid addition for shape stability in molded foods [49]. Compared with molded foods, 3D printing one can create delicate food structures with non-gelatinous texture. Besides, 3D printed dysphagia foods may include less handling, less amount of plastic used and less storage space for consumption, which has been a concern for mold-shaped foods in long-term-care homes [9,49]. Additionally, 3D printing food enables the creation of intricate designed internal structure with customized texture through varying infill patterns and infill levels, which is not easy by molding [15]. We believe that, very soon, 3D printing would provide a feasible option for the dysphagic patients with enhanced appetite by creating attractive and appealing structure and pleasant texture.

### 3.6. 3D Printing Behavior

**Figure 8:**
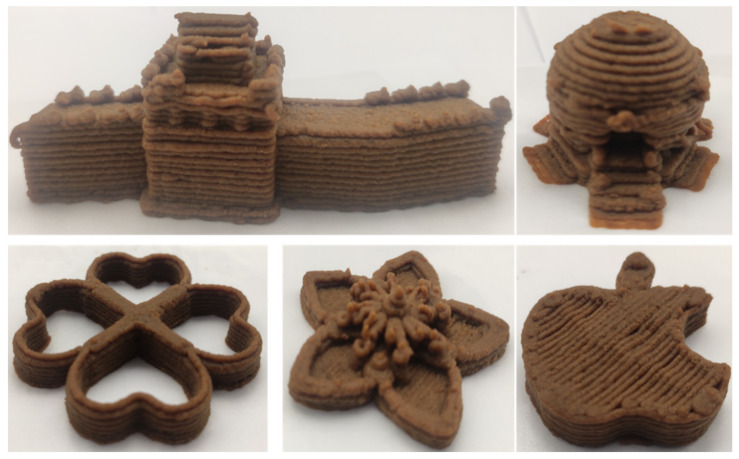
Pictures of 3D printed representative samples using XG-0.6% containing ink.

The ink samples were 3D printed into a hollow cylinder shape to assess their printability with different formulation. Generally, the samples printed with different formulations were quite close to the designed geometry after deposition. However, a slight deformation was observed over time for the cylinder printed with AG containing and control ink samples, indicated by a fatter bottom than upper part after storing for 60 min (Table 4). This was because that AG containing and control ink samples had relative low G′ and weak yield behavior (Figure 1 and Figure 3), so the printed samples were not strong enough to be self-supportable to resist gravity compressed deformation. Previous studies also reported that G′ and yield stress were important for the samples’ self-supporting capability [17]. The poor self-supporting performance of AG and control sample was also reflected by the relatively large creep % strain (Figure 6). The XG containing printed samples illustrated a smooth and appealing surface texture and no compressed deformation was observed. This was probably because the rendered creamy effect by XG through coating performance and enhancement of mechanical strength indicated by the improved G′ and yield stress (Figure 1 and Figure 3). Although the KG addition significantly increased the mechanical strength (G′, yield stress), KG containing printed samples showed a rough and dry surface structure with several defective points. Sometimes broken extrudate thread during printing was observed. This was probably because KG addition resulted in the formation of rigid and brittle structure [50] and significantly increased the mechanical strength of KG containing ink sample as indicated by the highest G′ and yield stress (Figure 1 and Figure 3). Figure 8 illustrates several printed samples using ink containing 0.6% XG. It was seen that samples with fine resolution and smooth surface displayed an attractive shape and visual appeal. This can certainly increase the appetite of people with swallowing disorders and increase the pleasure of eating.

## 4. Conclusions

The aged population is increasing rapidly with the growth of aging society. It is a great challenge to provide the swallow-impaired elderly with feasible diets due to lack of nutritious appetizing food options. 3D food printing allows for the nutrition personalization of regular-natural food structure with visual appeal at given special mealtime needs. This study investigated the potential to produce dysphagic diets using shiitake mushroom by 3D printing with addition of KG, XG and AG. The mechanical strength of self-supporting capacity of 3D printed samples were enhanced when KG and XG were added. The KG and XG containing samples were classified as level 5—minced and moist dysphagia diet within IDDSI framework, except KG-0.6% and KG-0.9% sample as they were too sticky for levels of 4 and 5. AG addition decreased the viscosity and mechanical strength of ink samples, and the resultant self-supporting capacity of 3D printed samples. Generally, the XG containing samples showed fine 3D printability with appealing appearance and were feasible for the production of dysphagia diets. This study provides insights for the development of dysphagia diets by emerging 3D printing technology, which would offer more options for the swallow- or chew-impaired patients.

## Figures and Tables

**Table 1:** Description of characteristics of dysphagia diets of level 4 and 5.

Classification	Level 4 (Pureed)	Level 5 (Minced & Moist)
Description	Usually eaten with a spoon (a fork is possible); Cannot be drunk from a cup because it does not flow easily; Cannot be sucked through a straw; Does not require chewing; Can be piped, layered or molded because it retains its shape, but should not require chewing if presented in this form; Shows some very slow movement under gravity but cannot be poured; Falls off spoon in a single spoonful when tilted and continues to hold shape on a plate; No lumps; Not sticky; Liquid must not separate from solid;	Can be eaten with a fork or spoon; Could be eaten with chopsticks in some cases, if the individual has very good hand control; Can be scooped and shaped (e.g., into a ball shape) on a plate;Soft and moist with no separate thin liquid; Small lumps visible within the food; Lumps are easy to squash with tongue;

**Table 2:** Viscosities at the shear rates of 1 s^−1^ (η_1_) and 10 s^−1^ (η_10_), and power-law fitted parameters of ink samples.

Formulations	*η*_1_	*η*_10_	Pow Law		
			*K* (Pa·s^n^)	*n*	R^2^
Control	336.85 ± 3.04 ^c^	62.54 ± 12.25 ^ab^	361.37 ± 1.52 ^c^	0.19 ± 0.01 ^d^	0.998
AG-0.3%	313.75 ± 0.49 ^b^	55.76 ± 5.01 ^a^	347.17 ± 0.79 ^b^	0.17 ± 0.01 ^d^	1.000
AG-0.6%	289.40 ± 9.81 ^a^	53.79 ± 2.69 ^a^	316.53 ± 9.63 ^a^	0.16 ± 0.00 ^c^	0.999
AG-0.9%	273.30 ± 28.26 ^a^	53.25 ± 6.98 ^a^	305.69 ± 25.97 ^a^	0.14 ± 0.05 ^b^	0.999
XG-0.3%	377.93 ± 11.43 ^d^	66.52 ± 8.23 ^ab^	400.32 ± 16.52 ^d^	0.18 ± 0.01 ^d^	0.998
XG-0.6%	380.30 ± 9.19 ^d^	61.92 ± 4.68 ^ab^	408.56 ± 14.46 ^d^	0.11 ± 0.03 ^b^	0.999
XG-0.9%	402.87 ± 63.56 ^e^	53.41 ± 1.56 ^a^	469.94 ± 34.00 ^e^	0.06 ± 0.01 ^a^	1.000
KG-0.3%	600.13 ± 64.84 ^f^	90.85 ± 7.76 ^c^	670.97 ± 92.96 ^f^	0.14 ± 0.01 ^bc^	0.999
KG-0.6%	1539.50 ± 82.73 ^g^	165.25 ± 5.30 ^d^	1691.01 ± 89.26 ^g^	0.12 ± 0.01 ^b^	0.999
KG-0.9%	2122.33 ± 339.65 ^g^	191.23 ± 26.11 ^e^	2242.43 ± 340.16 ^h^	0.10 ± 0.01 ^b^	0.999

Note: Data was reported as average ± standard deviation. Different lowercase letters indicate significant differences.

**Table 3:** Categorization of samples using IDDSI evaluation methods.

Sample	Fork Pressure Test	Fork Drip Test	Spoon Tilt Test	Comments
Control	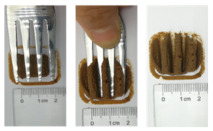	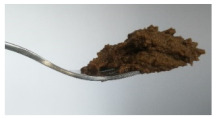	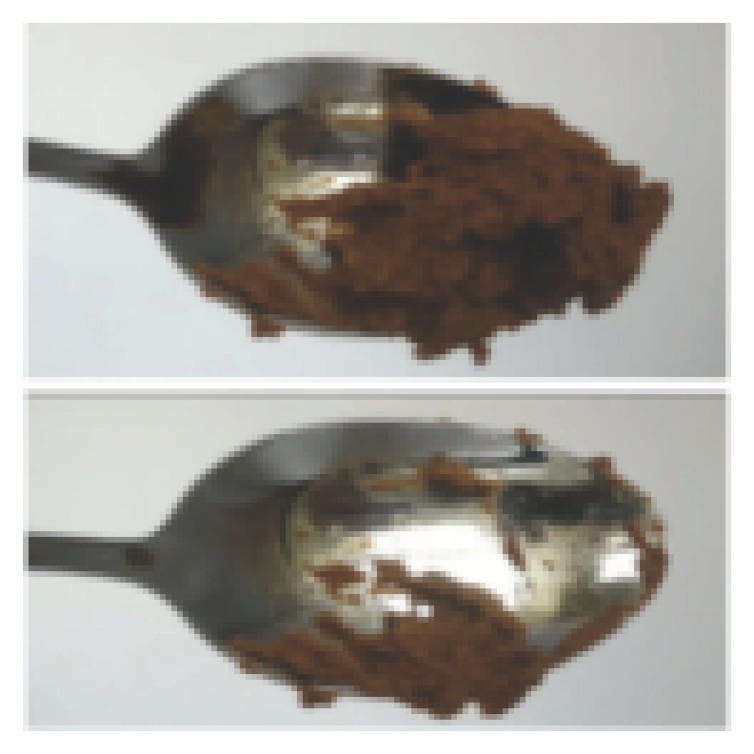	Level 5-Minced and moist
	Description: easily mashed with no lumps and granulation; a clear pattern was made on sample surface; no nail blanches white; no recovery of the deformed shape.	Description: sample piled above the fork; no sample form a short tail below the fork.	Description: sample was cohesive enough to hold its shape on the spoon; easily slide off the spoon with a very gentle flick; little food left on the spoon.	
AG-0.3%	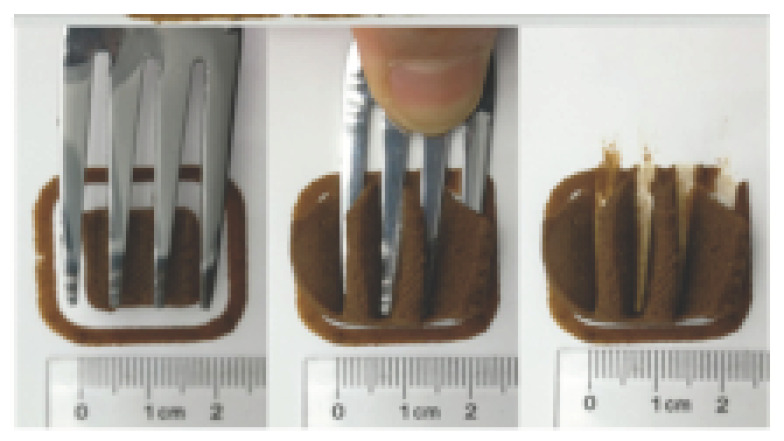	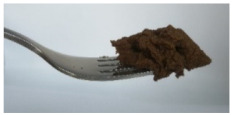	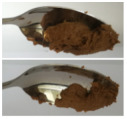	Not classified
	Description: easily separated between the prongs of a fork; a clear pattern was made on sample surface; no lumps and granulation; no recovery of the deformed shape.	Description: sample piled above the fork; no sample flow through or form a short tail below the fork.	Description: sample was cohesive enough to hold its shape on the spoon; not easy to slide off the spoon is titled, with lots of food left on the spoon.	
AG-0.6%	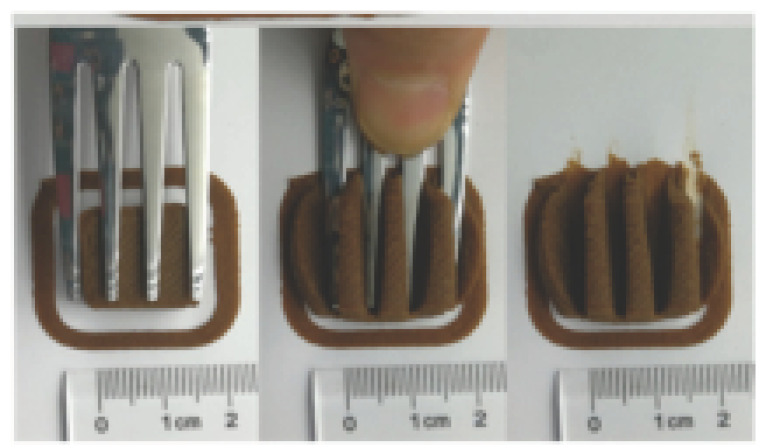	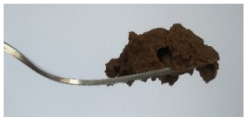	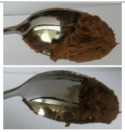	Not classified
	Description: easily separated between the prongs; a clear pattern was made; no lumps and granulation; no recovery of deformed shape when removing the fork.	Description: sample piled above the fork; no sample flow through or form a short tail below the fork.	Description: cohesive enough to hold its shape on the spoon; not easy to slide off when the spoon is titled, with lots of food left on the spoon.	
AG-0.9%	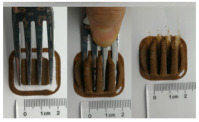	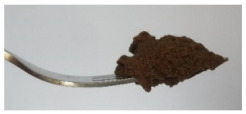	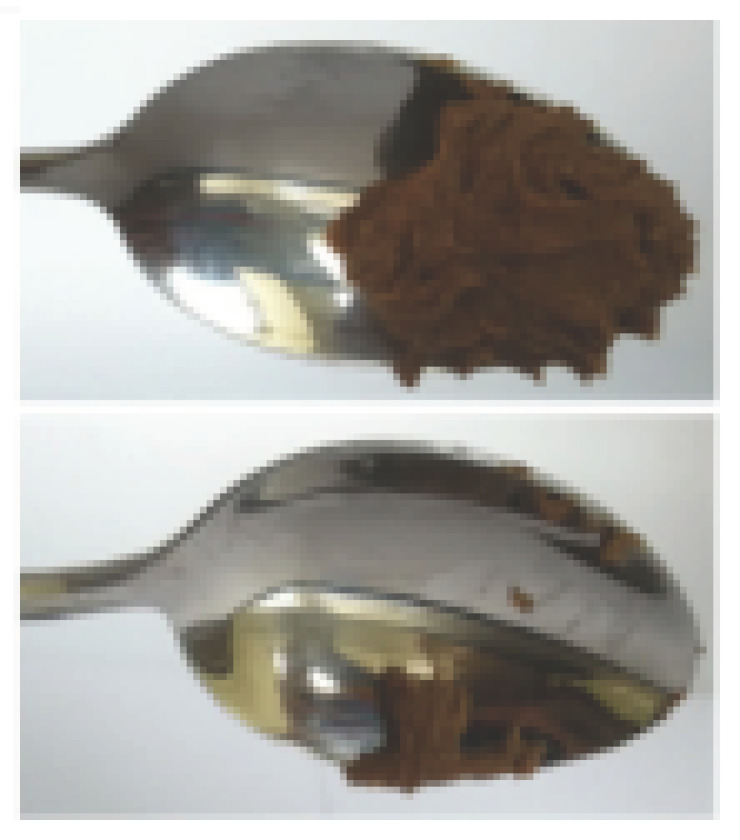	Level 5-Minced and moist
	Description: easily separated between the prongs of a fork; a clear pattern was made on sample surface; no lumps and granulation; no recovery of the deformed shape.	Description: sample piled above the fork; no sample flow through or form a short tail below the fork.	Description: sample was cohesive enough to hold its shape on the spoon; easily slide off the spoon with a very gentle flick; little food left on the spoon.	
XG-0.3%	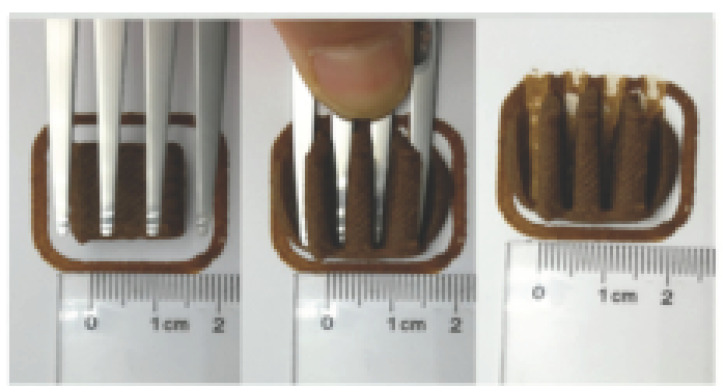	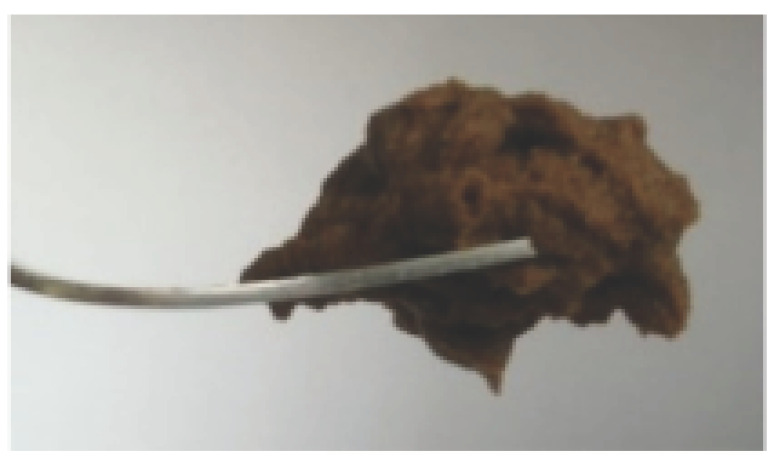	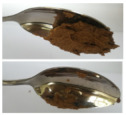	Level 5-Minced and moist
	Description: easily separated between the prongs of a fork; a clear pattern was made on sample surface; no lumps and granulation; no recovery of the deformed shape.	Description: sample piled above the fork; no sample flow through the fork.	Description: sample was cohesive enough to hold its shape on the spoon; easily slide off the spoon with a very gentle flick; little food left on the spoon.	
XG-0.6%	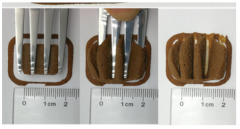	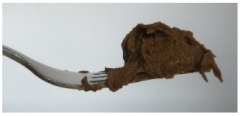	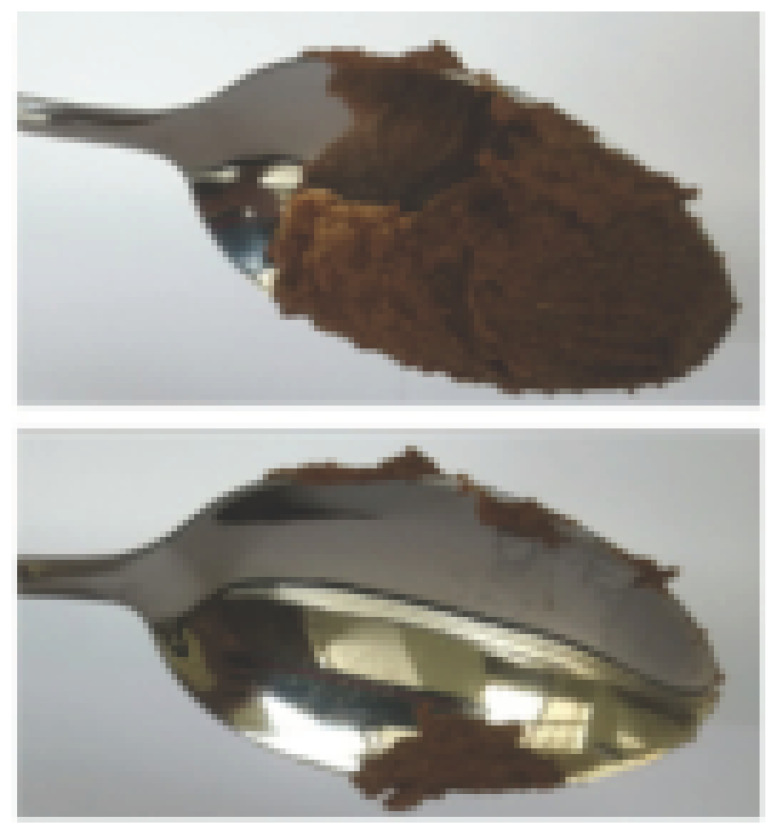	Level 5-Minced and moist
	Description: easily separated between the prongs of a fork; a clear pattern was made on sample surface; no lumps and granulation; no recovery of the deformed shape.	Description: sample piled above the fork; no sample flow through the fork.	Description: sample was cohesive enough to hold its shape on the spoon; easily slide off the spoon with a very gentle flick; little food left on the spoon.	
XG-0.9%	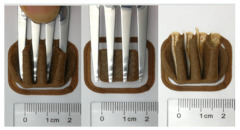	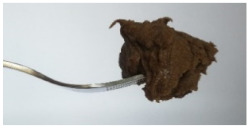	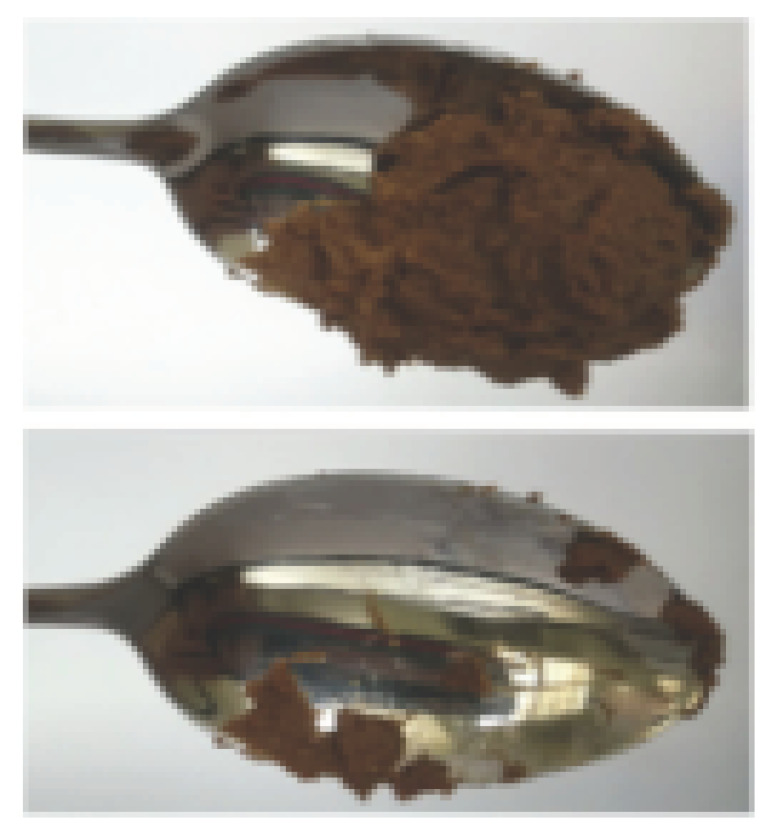	Level 5-Minced and moist
	Description: easily separated between the prongs of a fork; a clear pattern was made on sample surface; no lumps and granulation; no recovery of the deformed shape.	Description: sample piled above the fork; no sample flow through the fork.	Description: sample was cohesive enough to hold its shape on the spoon; easily slide off the spoon with a very gentle flick; little food left on the spoon.	
KG-0.3%	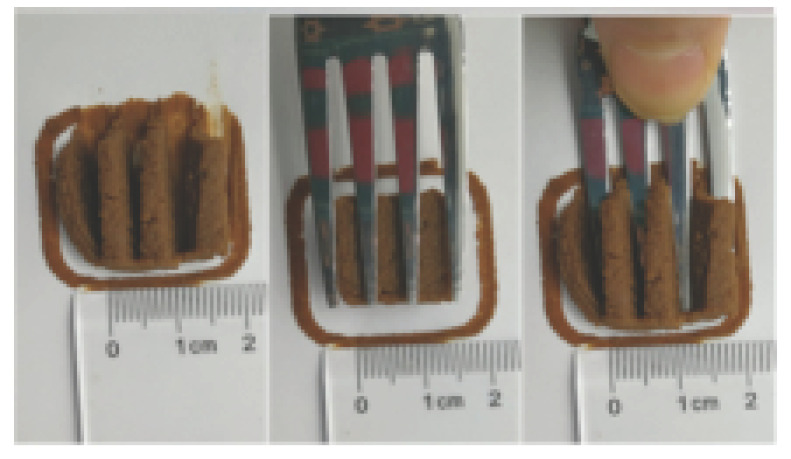	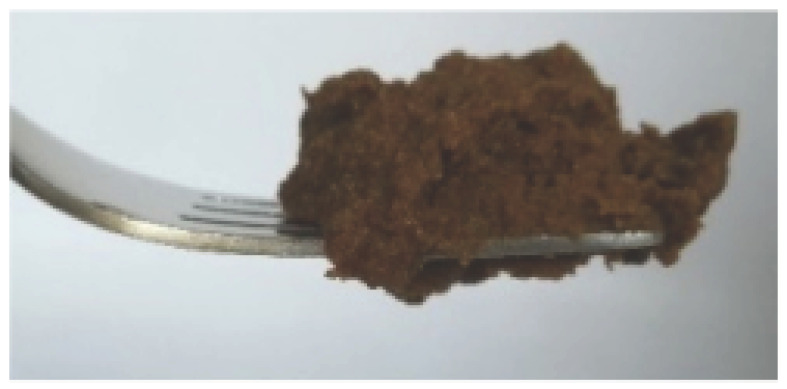	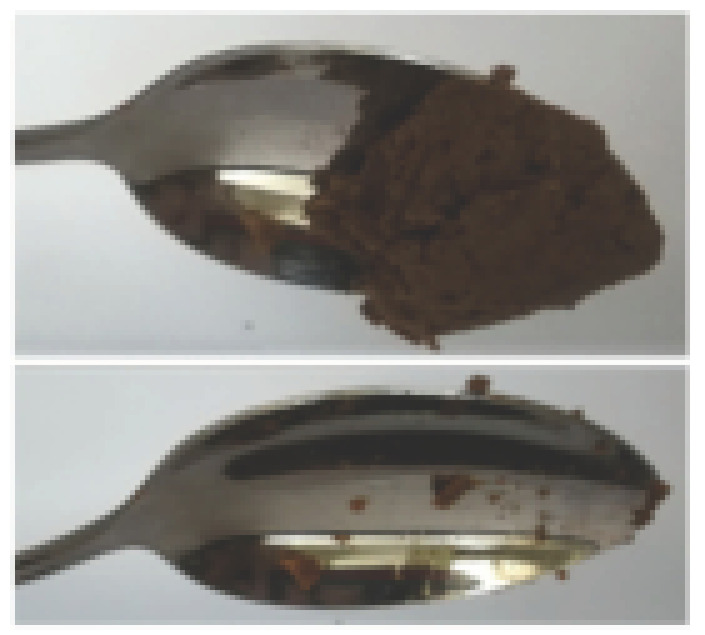	Level 5-Minced and moist
	Description: easily separated between the prongs of a fork; a clear pattern was made on sample surface; no lumps and granulation; no recovery of the deformed shape.	Description: sample piled above the fork; no sample flow through or form a short tail below the fork.	Description: sample was cohesive enough to hold its shape on the spoon; easily slide off the spoon with a very gentle flick; little food left on the spoon.	
KG-0.6%	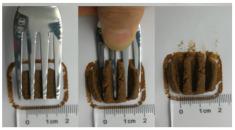	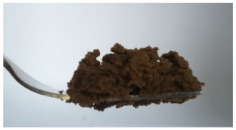	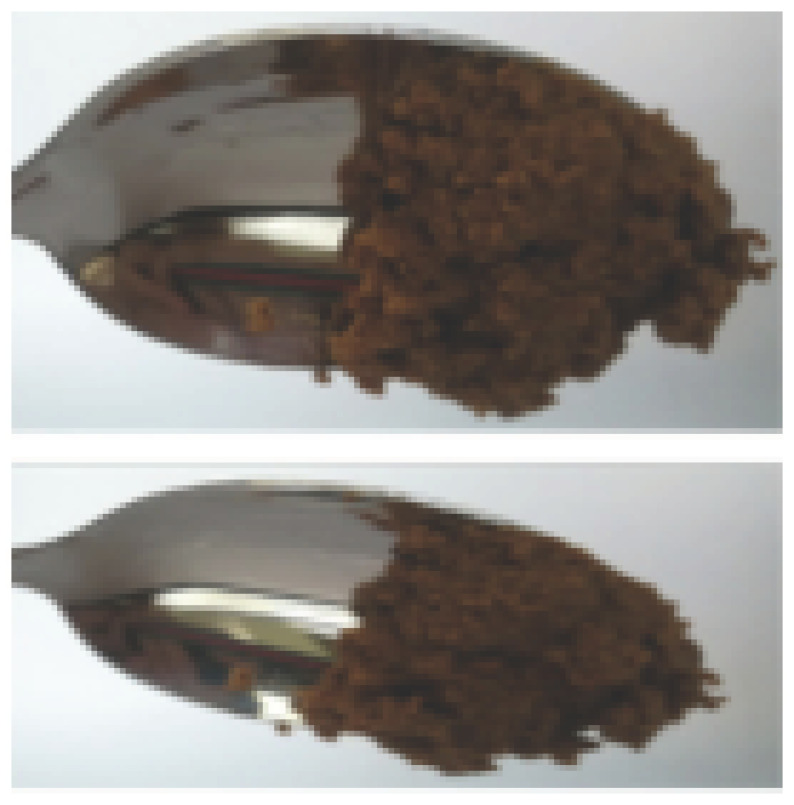	Not classified
	Description: easily separated between the prongs of a fork; a clear pattern was made on sample surface; no lumps and granulation; no recovery of the deformed shape.	Description: sample piled above the fork; no sample flow through or form a short tail below the fork.	Description: rough grainy structure; not easy to slide off when the spoon is titled, with lots of food left on the spoon.	
KG-0.9%	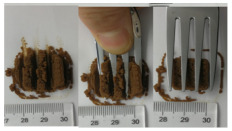	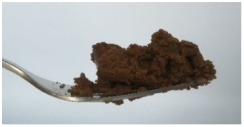	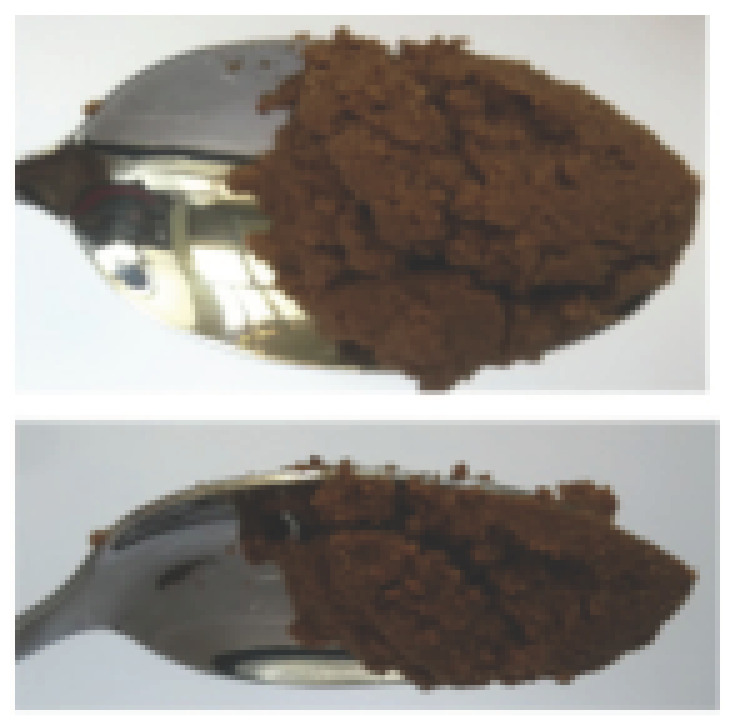	Not classified
	Description: easily mashed with little pressure; no blanch to white of thumb nail; smooth with no lumps; does not return to orginal shape when removing the fork.	Description: sample piled above the fork; no sample flow through or form a short tail below the fork.	Description: rough grainy structure; not easy to slide off when the spoon is titled, with lots of food left on the spoon.	

**Table 4:** The 3D printed samples with different formulation and storage time.

Time after Printing	Control			AG		
				0.3%	0.6%	0.9%
0 min	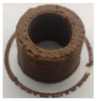	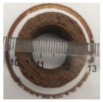	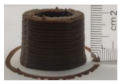	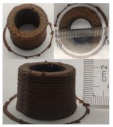	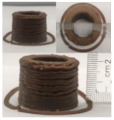	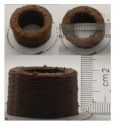
30 min	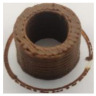	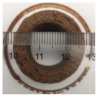	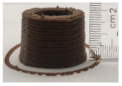	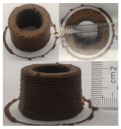	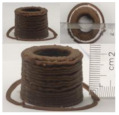	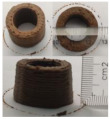
60 min	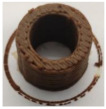	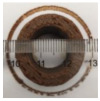	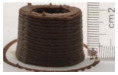	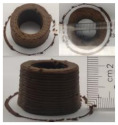	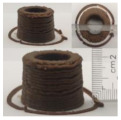	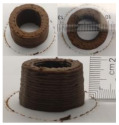
**Time after Printing**	**XG**			**KG**		
	0.3%	0.6%	0.9%	0.3%	0.6%	0.9%
0 min	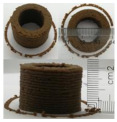	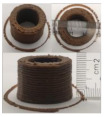	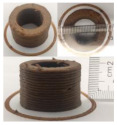	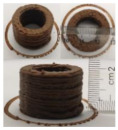	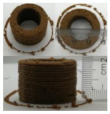	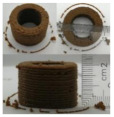
30 min	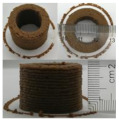	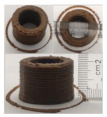	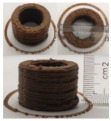	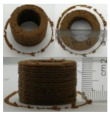	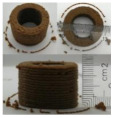	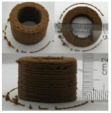
60 min	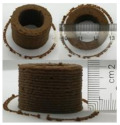	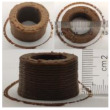	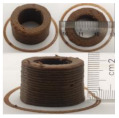	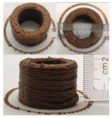	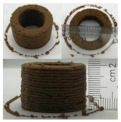	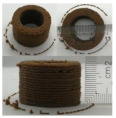

## Data Availability

The data presented in this study are available on request from the corresponding author.
